# Early onset frontotemporal dementia following cannabis abuse: a case report

**DOI:** 10.1186/s12888-023-04956-w

**Published:** 2023-06-30

**Authors:** Reza Moshfeghinia, Bahare Oji, Mehrnaz Hosseinzadeh, Mohammad Pourfridoni, Jamshid Ahmadi

**Affiliations:** 1 Student Research Committee, Shiraz University of Medical Sciences, Shiraz, Iran; 2 Research Center for Psychiatry and Behavioral Sciences, Shiraz University of Medical Sciences, Shiraz, Iran; 3 Substance Abuse Research Center, Shiraz University of Medical Sciences, Shiraz, Iran; 4 Fasa Neuroscience Circle (FNC), Student Research Committee, Fasa University of Medical Sciences, Fasa, Iran; 5 Student Research Committee, Jiroft University of Medical Sciences, Jiroft, Iran; 6 Substance Abuse and Mental Health Research Center, Shiraz University of Medical Sciences, Shiraz, Iran; 7 Institute for Multicultural Counseling & Education Services (IMCES), Los Angeles, CA US

**Keywords:** Frontotemporal dementia, Cannabis, Addiction, Dementia

## Abstract

**Background:**

Frontotemporal disorders (FTD) are the consequence of impairment to neurons in the frontal and temporal lobes of the brain. Also, no definitive treatment has been found for FTD. Cannabinoid products can be used to manage treatment-resistant behavioral variants of Frontotemporal dementia (bvFTD).

**Case presentation:**

We describe the case of 34 years old male with two years of marijuana abuse. At first, he presented with symptoms of apathy and bizarre behavior, which became more severe, and led to disinhibition. The clinical symptoms and imaging findings made FTD probable for him, which was very interesting to report.

**Conclusions:**

While cannabis has demonstrated potential in managing behavioral and mental symptoms of dementia, the presented case highlights the profound impact of cannabis consumption on brain structure and chemistry, including the potential for neurodegenerative disorders like FTD.

## Background

Frontotemporal dementia (FTD) is the consequence of damage to neurons in the frontal and temporal lobes of the brain, which is characterized by progressive deficits in behavior, executive function, or language. The disease can mimic many psychiatric disorders because of the prominent behavioural features [1].

FTD is one of the most common types of dementia before 65 years of age [2] and rarely occurs in individuals under 30 [3]. FTD, onset before age 30, tends to show frequent sudden changes in mood, behavioral disinhibition, increased aggression, decreased empathy, and problems in working memory [4].

So far, no definitive treatment has been found for FTD, and current treatments are based on controlling symptoms and reducing their severity. Studies have shown that antidepressants and second-generation antipsychotics may help these patients [5]. Cannabinoids are one of the potential agents under investigation for symptom reduction of dementia. Several studies have shown that prescribing cannabinoid products improves FTD patients’ behaviors and initial symptoms [6, 7]. Although some studies have shown that cannabinoids are effective in improving the initial symptoms of FTD, some studies also show that starting to use cannabinoids from the beginning of adulthood and its long-term use is related to hippocampal atrophy and poor cognitive performance in middle age and it is a known risk factor for dementia [8–10].

This report discusses a case that developed early-onset FTD at age 34 following cannabinoid use. The items that make this case rare and reportable are the occurrence of FTD at a young age and its occurrence following the use of cannabinoids. At the same time, several studies have shown the short-term positive effects of cannabinoids on symptom reduction FTD.

## Case presentation

A 34 years old man without any significant previous psychiatric history except for marijuana abuse for the last two years, with normal neurodevelopment throughout his life, had his first medical encounter due to his family’s complaint of restlessness and a significant decline in social activities. His family reported that he was socially withdrawn and didn’t go to work recently. Other symptoms included anhedonia, apathy, and a significant decline in all areas of functioning. He was admitted to the psychiatric hospital. After mental status examination and history taking, an antidepressant was started for the patient. Finally, the patient was discharged after partial improvement (48 days) with the diagnosis of marijuana amotivational syndrome.

**Fig. 1:**
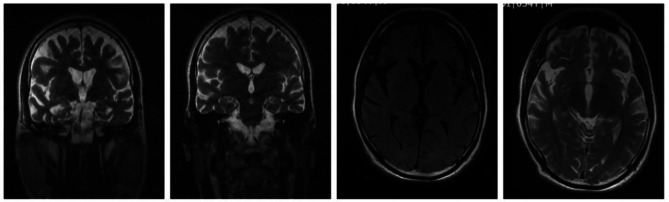
Brain magnetic resonance imaging (MRI): Frontotemporal lobar degeneration and cortical brain atrophy are more significant in the frontal, parietal and temporal lobes

According to the patient’s father, the patient’s symptoms worsened after the discharge. During the follow-up, the patient was referred to several doctors (neurologist and psychiatrist), and magnetic resonance imaging (MRI) was requested for him. MRI displayed Frontotemporal lobar degeneration, with cortical brain atrophy more significant in the frontal, parietal, and temporal lobes (Fig. 1). Due to the non-specific symptoms, a definite diagnosis was not given to the patient, and only brain atrophy was explained to the patient’s father.

Two years after the onset of the first symptoms and hospitalization, the patient was brought into the emergency department (ED) with various progressive psychiatric symptoms. The patient had perseverative behaviors without obsession, as he bathed about ten times a day and performed long walking to count buildings and other objects in his neighborhood. He also suffered from disinhibition in such a way that he borrowed money from people around him without any purpose. Bizarre behaviors such as defecation, urination in the street, and worsening of amnesia from about a year ago have progressed to Anomia and prosopagnosia. The patient had hyperorality, as he placed both edible and inedible objects in his mouth, and finally, the patient suffered from apraxia and echolalia. He had symptoms of irritability, mutism, disorganized behaviors, increased appetite, memory impairment, urinary and fecal incontinence, and lack of pain perception. He also had anosognosia, did not demonstrate any insight into his life problems, including his divorce, and declined any psychiatric or medical problems. This presentation led to the second admission of the patient, antipsychotic was started for him, showing some benefits.

**Fig. 2:**
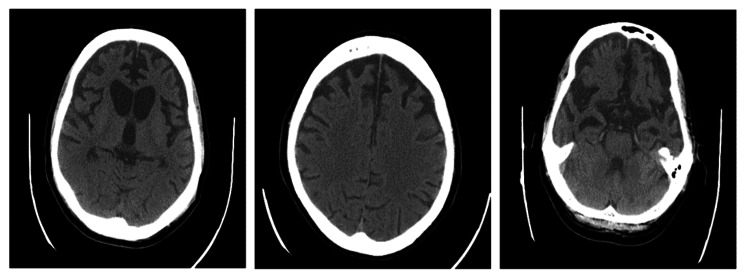
computed tomography (CT) scan: Cortical brain atrophy is more significant in the frontal, parietal, and temporal lobes. Dilatation of both lateral ventricles, especially in the frontal horn and third and fourth ventricles, suggests focal atrophy

For a workup of this atypical presentation of psychosis, and associated symptoms, a brain computed tomography (CT) scan was taken. Non-contrast spiral brain CT scan through the brain without gadolinium contrast demonstrates bilateral cortical brain atrophy inappropriate for the patient’s age, which was more in the frontal, parietal and temporal lobes and is suggestive of frontotemporal lobar degeneration (Fig. 2).

His family history was extensively reviewed except for one second-degree relative with a suicidal attempt and another second-degree relative with an unknown psychiatric illness requiring him to undergo Electroconvulsive therapy. No familial history of dementia, cognitive impairment, parkinsonism, and other neurodegenerative disease was noted.

Due to the early-onset presentation and severe behavioral and personality changes, lack of previous psychiatric or medical history, lack of specific stressors or risk factors, lack of familial history of neurodegenerative disease, and brain imaging findings with prominent cortical brain atrophy, especially in frontal, parietal and temporal lobes, early-onset FTD post cannabinoids consumption was considered as the final diagnosis.

## Discussion and conclusion

We present a progressive case of early-onset frontotemporal dementia without significant past medical history except for Marijuana use for two years before the onset of symptoms. The diagnosis was made according to the typical presentation of almost all core features of the probable bvFTD, including behavioural disinhibition, apathy, loss of empathy, compulsive behaviours, hyperorality, and the inevitability of quitting his job due to deficit in executive functions. Our patient also showed significant insensitivity to pain [1]. Brain imaging studies indicated bilateral significant frontotemporal lobar atrophy inappropriate for his age.

Intuitive attitude and a common misconception about the early-onset presentation of dementia bring up genetic inheritance as the cardinal etiology. Nevertheless, plenty of observational studies show that only a minority of early-onset dementia, including both Alzheimer’s disease (AD) and FTD, have autosomal dominant patterns or can be accredited to known gene mutations [11–13]. When multiple relatives experience FTD or a comparable illness through successive generations, it is considered to be familial FTD. As an example, a grandparent, parent, and adult child are all closely related to one another and all belong to one side of the family, not both. When only one member of a family develops FTD, it is considered to be sporadic FTD. Hence, there are deliberate endeavors to identify the environmental and lifestyle risk factors contributing to the disease presentation; Regarding early-onset FTD, occupational exposure to heavy metals, metalloids, dyes, pesticides, along with smoking, heavy alcohol consumption, traumatic brain injuries, and traumatic professional sports, obesity, and particularly cumulative exposure to risk factors are among the known environmental and modifiable risk factors [13–15]. Air pollutants, occupational exposures, cardiovascular risk factors, and dietary consumption of cereal, dairy, dry cake, and ice cream are shown to be associated with an increase in the odd ratio of disease presentation [12, 16].

Considering our presenting case, the lack of significant familial history, the insufficiency of possible attributable risk factors, and the significant history of Marijuana consumption before symptom onset suggest us impute the connection between cannabis and early-onset FTD. A likely hypothesis is that as an illicit drug in the country without standardized preparation and processing, cannabis users are at high risk of receiving cannabis contaminants and toxins in unsafe quantities. Common cannabis contaminants are heavy metals, pesticides, and microbes [17] that can increase the risk of FTD [13, 14].

A large number of previous studies on the usage of cannabis indicate its promising effects on symptom control of behavioural and psychological symptoms of dementia (BPSD). Anti-oxidant, anti-neuroinflammatory effects, increase in the neurogenesis activation, and pro-survival factors including Brain-Derived Neurotrophic Factor (BDNF) and inhibition of tau hyperphosphorylation are among the contributing mechanisms [18, 19].

Most studies examining the effect of cannabis on controlling BPSD involve AD patients or did not specify the type of dementia; in our literature review, only a few studies specifically present the effect of cannabis on FTD patients, and together they introduced nine FTD patients. Gopalakrishna et al. [6], in a case series including three FTD cases showed that cannabinoids could improve behavioural symptoms and ameliorate caregiver burden [6]. Another case series of 17 cases encompassed three FTD patients; two reported significant improvements in behaviour and mood 18. Amanullah et al. [20], Pia-Escudero et al. [21], and Zajac et al. [22] each present a case of FTD (or mixed FTD and vascular dementia) with positive results, including improvement in agitation, behavioural disinhibition, bruxism, and increased sociability.

Almost all the studies investigating the effect of cannabis and cannabinoid on BPSD were not contesting the long-term efficacy or the adverse effects of chronic marijuana use in dementia. They were conclusive based on the promising short-term results [18]. However, looking into the brain of chronic cannabis users would illustrate its deleterious effects. As in a study by Amen et al., persistent hypoperfusion in multiple brain regions, including the hippocampus, parahippocampal gyrus, precuneus, posterior cingulate, and medial temporal lobe, was significantly shown [23]. The orbitofrontal cortex and hippocampus are the most affected brain regions through studies [24]. The large cohort of the IMAGEN study demonstrates the negative association between adolescent marijuana abuse and cortical thickness in the prefrontal cortex [25]. Cannabis consumption is related to reduced gray matter volume in the medial temporal cortex, insula, temporal pole, orbitofrontal cortex, and parahippocampal gyrus; Regions rich in cannabinoid CB1 receptors and functionally related to emotional, motivational, and affective processing [26]. Another impaired process in the brain of chronic marijuana users is the dopamine deficit, which can also make the picture of FTD pathology [27, 28].

Cannabinoid use would impair the regulation of endocannabinoid system in its critical stage of development, Renard et al. showed that the chronic cannabinoid exposure during adolescence has been shown to induce long-term changes in the PFC, such as reduced dendritic arborization of pyramidal neurons, impaired synaptic plasticity, altered expression of synaptic markers, decreased gray matter volume, and abnormal connectivity. These changes may impair the communication between the PFC and other brain regions, such as the hippocampus, which is essential for learning and memory. As a result, chronic cannabinoid exposure during adolescence may increase the risk of developing frontotemporal dementia later in life [29].

The duration of cannabis use that can cause neurocognitive problems is not clearly determined in the literature. However, some studies have suggested that chronic cannabis use, defined as regular consumption for at least one year with a minimum frequency of once per week, is associated with significant impairments in cognitive impulsivity, cognitive flexibility, attention and short-term memory [30], and verbal learning and memory [31]. Furthermore, the age of onset of cannabis use and the quantity and potency of cannabis consumed may also influence the neurocognitive effects, [30, 31]. Therefore, it is likely that the longer, earlier and more frequently someone uses cannabis, especially high-potency strains, the more severe and persistent the neurocognitive problems will be.

Since cannabis has a complex pharmacology, medical use demands extensive formulary studies. The two most prominent constituents of cannabis, delta-9-tetrahydrocannabinol (THC) and cannabidiol (CBD), have several paradoxical and sometimes confronting effects. THC, as the main psychoactive factor of cannabis, is shown to cause cognitive dysfunction, psychosis, and alteration in cortical structure and function in adolescents [32, 33]. CBD has a lower risk profile and is accounted to alleviate THC’s harmful effects. It is regarded to act as an antipsychotic, anxiolytic, and immune modulator. However, due to the limitation of evidence, CBD prescription for psychiatric disorders is still an open debate [33]. It has been shown that there is no information on the CBD content for more than 58% of cannabinoid products, and the amount of side effects of these products increases with the ratio of THC to CBD [34]. Recreational and illegal use of cannabis, as in our patient, are at risk of adverse effects due to formulary reasons.

These severe effects on the brain structure and brain chemistry of chronic cannabis users may potentially contribute to the development of neurodegenerative disorders like FTD in genetically vulnerable individuals. While the warning about cannabis consumption’s impact on brain structure is valid, the scarcity of reported FTD cases in patients with chronic cannabis use suggests the need for more nuanced conclusions. Therefore, the recommendation of cannabis as medical management for BPSD should be approached with additional caution, particularly for early-onset patients who are more likely to engage in long-term use and dependence, increasing their vulnerability to neurodevelopmental abnormalities.

## Data Availability

Not applicable as this is a case report.

## Abbreviations

FTD: Frontotemporal disorders
bvFTD: Behavioral variants of Frontotemporal dementia
MRI: Magnetic resonance imaging
ED: Emergency department
CT: Computed tomography
BPSD: Behavioural and psychological symptoms of dementia
BDNF: Brain-Derived Neurotrophic Factor
AD: Alzheimer’s disease
THC: Delta-9-tetrahydrocannabinol
CBD: Cannabidiol
